# GADD34 inhibits activation-induced apoptosis of macrophages through enhancement of autophagy

**DOI:** 10.1038/srep08327

**Published:** 2015-02-09

**Authors:** Sachiko Ito, Yuriko Tanaka, Reina Oshino, Keiko Aiba, Suganya Thanasegaran, Naomi Nishio, Ken-ichi Isobe

**Affiliations:** 1 Department of Immunology, Nagoya University Graduate School of Medicine, 65 Turumai-cho, Showa-ku, Nagoya, Aichi, 466-8550, Japan

## Abstract

Autophagy is a common physiological function in all eukaryotes. The process is induced by depletion of nutrients including amino acids. GADD34 is expressed following DNA damage, ER stresses and amino acid deprivation. Here, we investigated the effects of GADD34 on autophagy and cell activation in macrophages. The deprivation of tyrosine and cysteine markedly induced the expression of GADD34 in macrophages. LPS stimulation combined with tyrosine/cysteine-deprivation initially activated macrophages, but then shifted to cell death in late phase of stimulation. When LPS stimulation was combined with tyrosine/cysteine-deprivation, a deficiency of GADD34 enhanced cell activation signaling such as Src-family, Erk1/2, p38 MAPK and Akt. In the late phase of stimulation, a deficiency of GADD34 increased apoptosis more than that in wild-type macrophages. Further we found that mTOR-S6K signaling was highly enhanced in GADD34-deficient macrophages compared with wild-type cells when cells were treated by LPS combined with tyrosine/cysteine-deprivation. LC3-II was increased by LPS stimulation combined with tyrosine/cysteine-deprivation. Defective GADD34 reduced LC3-II and autophagosome formation induced by LPS-stimulation and tyrosine/cysteine-deprivation compared with that seen in wild-type macrophages. These results indicates that GADD34 enhances autophagy and suppresses apoptosis stimulated by LPS combined with amino acid deprivation through regulation of mTOR signaling pathway in macrophages.

Autophagy is an intracellular degradation pathway essential for cellular and energy homeostasis. In the process of autophagy, cytosolic materials, including disused organelles, toxic aggregated proteins and pathogens are sequestered into membrane vesicles and then delivered to lysosomes for degradation^1^. Although autophagy was first discovered in mammalian cells^2^,^3^, the molecular mechanisms of autophagy were first elucidated by Ohsumi's group in the yeast *Saccharomyces cerevisiae* in 1992^4^. They found that nitrogen starvation and depletion of nutrients such as carbon and single amino acids induced autophagy in yeast. This finding accelerated the discovery of molecules involved in autophagy.

There are many homologies between autophagy and phagocytosis of microorganisms. Macrophages are professional phagocytic cells in mammals. Autophagy is strongly induced during nutrient starvation, and this state leads to bulk degradation of cytoplasmic components, the building blocks of which are used to supply energy and to synthesize components essential for survival under conditions of nutrient starvation^5^. Because autophagy digests cytosolic materials including pathogens sequestered in membrane vesicles, autophagy may be a primordial form of eukaryotic innate immunity against invading microorganism^6^.

Growth arrest and DNA damage-inducible protein (GADD34/Ppp1r15a) was originally isolated from UV-inducible transcripts in Chinese hamster ovary (CHO) cells^7^. Evidence suggests that GADD34 may be functional in myeloid cells including macrophages. For example, GADD34 was cloned from a radiation-treated hamster myeloblastic leukemic cell cDNA library^8^. Moreover, mouse myeloid differentiation primary response 116 (Myd116), which is equivalent to hamster GADD34, was cloned from M1 myeloblastic leukemia cells treated with IL-6 to induce terminal differentiation^9^. Expression of GADD34 is upregulated by growth arrest and DNA damage^10^. It is also induced by amino acid deprivation and several endoplasmic reticulum (ER) stresses^11^,^12^. We have shown that GADD34 suppresses signaling by mammalian target of rapamycin (mTOR)^13^,^14^, which is a central inhibitor of autophagy^15^. mTOR is a regulator of the translational machinery in response to cellular stress. mTOR occurs in two multiprotein complexes, mTOR complex 1 (mTORC1) and mTORC2. mTORC1 responds to insulin and amino acids to control growth and protein translation^16^,^17^. It controls cell growth through phosphorylation of p70 ribosomal protein S6 kinase and controls protein synthesis by modulating the activity of eukaryotic initiation factor 4-binding protein 1(4E-BP1)^18^.

Based on these prior studies, we hypothesized that GADD34 might work as an autophagy regulator in innate immune cells such as macrophages and dendritic cells. We have shown previously that starvation induces the expression of GADD34 and reduced mTOR activity *in vivo*^14^. In order to analyze the effects of GADD34 on more specific types of nutrient deficiency, we depleted specific amino acids from culture medium and stimulated macrophages with LPS to investigate the effects of GADD34 on autophagy in innate immune cells.

## Results

### Combined deprivation of tyrosine (Tyr) and cysteine (Cys) markedly induced GADD34 expression

**Figure 1:**
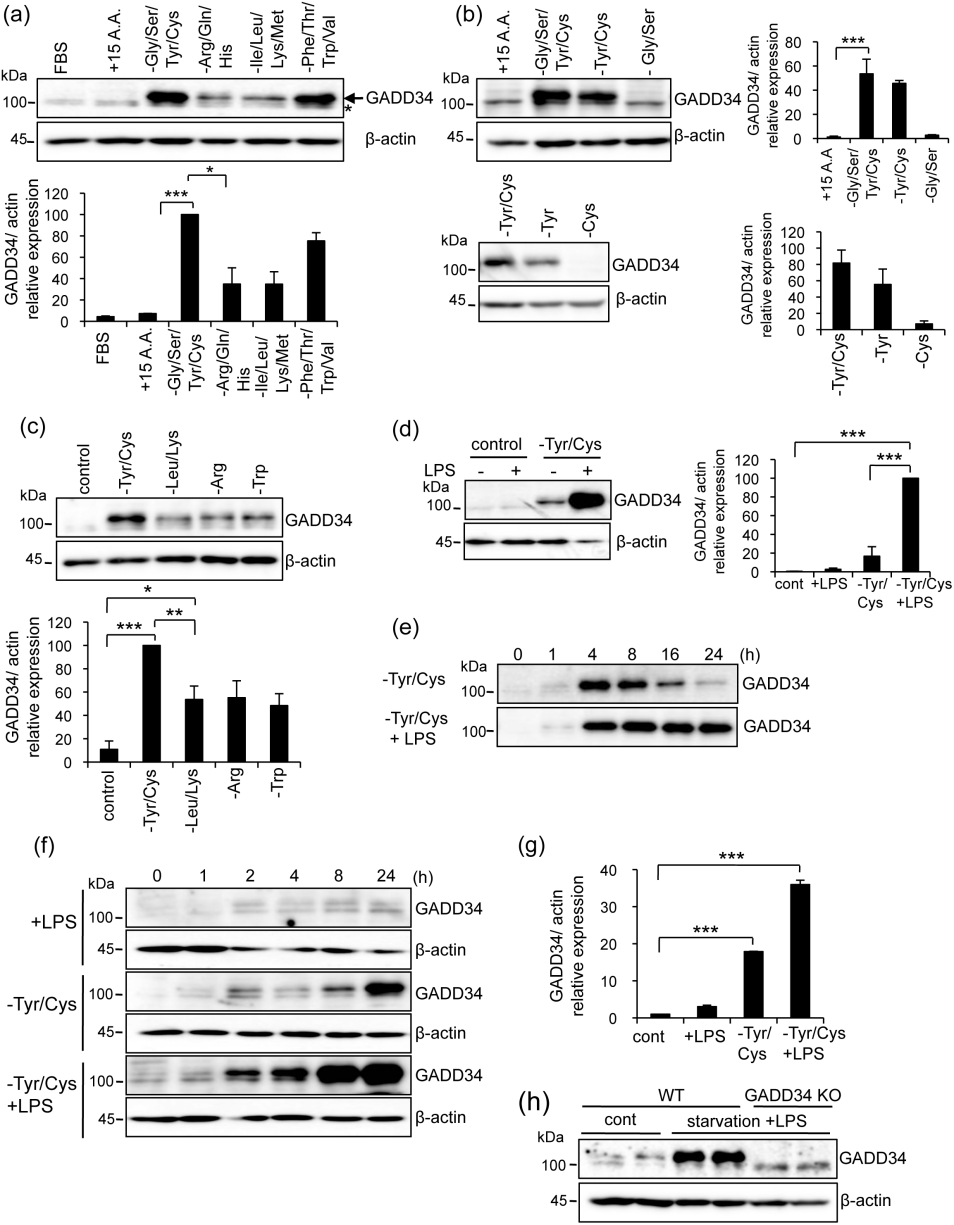
Expression of GADD34 induced by amino acid-deprivation and LPS. (a). Deprivation of grouped amino acids. RAW 264.7 cells were cultured with the indicated amino acid deprivation media for 4 h. Cell lysates were immunoblotted with anti-GADD34 and anti-β-actin antibodies. Β-actin was used as a control. Arrow indicates GADD34-specific bands. Asterisk indicates non-specific bands. Intensities of the bands were measured by densitometry. Graph shows the immunoblot relative intensities as means ± SE of 3 independent experiments. FBS; DMEM + 10% FBS, +15 A.A. (amino acid); Basal medium containing 15 amino acids (Table 1) without FBS. Gly/Ser/Tyr/Cys deprivation; Arg/Gln/His deprivation; Ile/Leu/Lys/Met deprivation; Phe/Thr/Trp/Val deprivation. (b). Deprivation of single or two amino acids. Assay was performed as in a. (c). Effects of amino acid deprivation. Expression of GADD34 was analyzed for Leu/Lys, Arg or Trp deprivation compared to Tyr/Cys deprivation. (d). RAW 264.7 cells were stimulated with LPS (1 μg/mL) in DMEM + 10% FBS (control) or Tyr/Cys-deprivation medium for 4 h. GADD34 expression was determined by immunobloting. (e). Time course (0–24 h) of GADD34-expression in RAW 264.7 cells incubated in Tyr/Cys-deprivation medium with or without LPS (1 μg/mL). (f). BMDMs were stimulated with LPS (1 μg/mL) in control or Tyr/Cys- deprivation medium for 0–24 h. (g). Human macrophage THP1 cells were stimulated with LPS, deprived of Tyr/Cys or both for 8 h. The expression of GADD34 was determined by real-time PCR. Β-actin was used as an internal control. (h). GADD34 KO or WT mice were starved and injected with LPS (5 μg/g body weight). Expression of GADD34 in bone marrow was analyzed after 24 h starvation and LPS stimulation. The original immunoblots are presented in Supplementary Figure 3. All immunoblots are representative of three independent experiments (a–f, h). Graph shows the relative expression as means ± SE of three independent experiments (a–d, g). *p < 0.05, **p < 0.01, ***p < 0.001.

In order to examine the effects of amino acid deprivation on GADD34 expression, we cultured RAW 264.7 macrophage cells with basal medium supplemented with 15 important amino acids (Table 1) in the absence of fetal bovine serum (FBS) for 4 h. When viable RAW 264.7 cells were examined, the expression of GADD34 was very low in this medium, the same as observed in DMEM containing 10% FBS (Fig. 1a lanes 1, 2). We then determined which amino acids induced the highest level of GADD34 expression when removed from the medium. We divided the amino acids into four groups and found that the deprivation of Gly/Ser/Tyr/Cys induced the highest level of GADD34 expression among them. Then, we compared the deprivation of Tyr/Cys and Gly/Ser compared to deprivation of Gly/Ser/Tyr/Cys. We found that Tyr/Cys deprivation but not Gly/Ser deprivation strongly induced GADD34 expression (Fig. 1b). Deprivation of Tyr alone but not Cys enhanced the level of GADD34 expression, although the expression level in the absence of Tyr was lower than that of Tyr/Cys deprivation (Fig. 1b). Because it had been shown previously that Leu deprivation induced GADD34 expression^19^ and Arg or Trp have important roles in innate immunity, we examined the effects of deprivation of Leu/Lys, Arg or Trp on GADD34 expression. Deprivation of these amino acids induced GADD34 expression (Fig. 1c). However, the expression level was lower than the Tyr/Cys deprivation.

To examine the function of GADD34 in activated macrophages, we stimulated RAW 264.7 macrophages with LPS to mimic a Gram-negative bacterial infection. LPS with Tyr/Cys-deprivation induced a much higher expression of GADD34 than did Tyr/Cys-deprivation alone (Fig. 1d). In RAW 264.7 cells, GADD34 expression peaked 4 h after the start of culture in Tyr/Cys-deprivation medium and then gradually decreased. In contrast, combining LPS treatment with Tyr/Cys-deprivation extended the expression of GADD34 for 24 h (Fig. 1e). Increase in GADD34 expression was also observed when bone marrow-derived macrophages (BMDMs) were exposed to LPS in combination with Tyr/Cys-deprivation (Fig. 1f), although GADD34 was slightly induced by LPS alone. Also in human macrophage cells THP1, the expression of GADD34 was significantly induced by LPS stimulation combined with Tyr/Cys-deprivation (Fig. 1g). *In vivo*, we observed the expression of GADD34 in bone marrow when mice were subjected to starvation and LPS-stimulation (Fig. 1h).

### GADD34 suppressed apoptosis induced by LPS stimulation combined with Tyr/Cys-deprivation in macrophages

**Figure 2:**
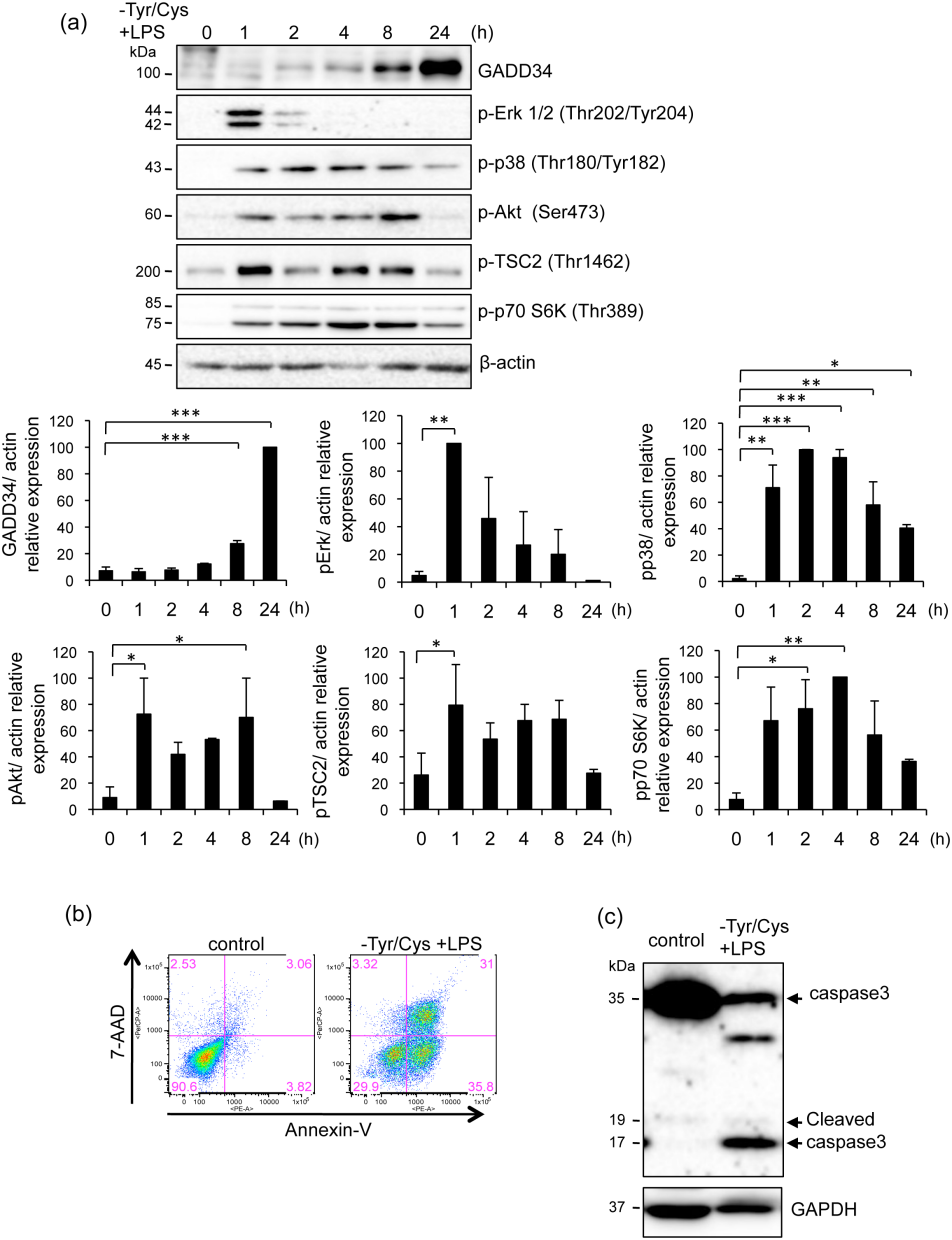
Activation-induced cell death induced by LPS stimulation combined with Tyr/Cys-deprivation in macrophages. (a). BMDMs derived from C57BL/6 mice were treated with LPS (1 μg/mL) with Tyr/Cys-deprivation for the indicated times (0–24 h). Cell lysates were immunoblotted with the indicated antibodies. Graph shows the relative expression as means ± SE of three independent experiments. (b). RAW 264.7 cells were treated with LPS (1 μg/mL) with Tyr/Cys-deprivation for 20 h. Cells were stained with 7-AAD and PE-labeled Annexin V and analyzed by flow cytometry. Data are representative of three independent experiments. (c). Immunoblotting of cleaved caspase 3 in RAW 264.7 cells treated with LPS (1 μg/mL) and Tyr/Cys-deprivation for 24 h. GAPDH was used as a control. Data are representative of three independent experiments. *p < 0.05, **p < 0.01, ***p < 0.001.

We examined the effects of amino acids deprivation combined with LPS stimulation of macrophages. LPS combined with Tyr/Cys-deprivation resulted in transient upregulation of cell growth-related signaling including p-Erk, p-p38, p-Akt, p-TSC2, and p-p70 S6K in BMDMs. They subsequently decreased concomitant with the increase in the expression of GADD34 (Fig. 2a). Similar results were obtained in RAW 264.7 cells (data not shown).

After cell activation by the stimulation of LPS combined with Tyr/Cys-deprivation, we observed that macrophage were gradually received damages. Thus we examined the cell damage under those conditions. Treatment of RAW 264.7 cells with LPS combined with Tyr/Cys-deprivation induced Annexin V^+^ 7AAD^−^ apoptotic cells and Annexin V^+^ 7AAD^+^ dead cells after 20 h (Fig. 2b). An apoptosis-related caspase 3 cleavage product also increased after stimulation (Fig. 2c). These results indicated that exposure to LPS combined with Tyr/Cys-deprivation induces activation-induced cell death.

**Figure 3:**
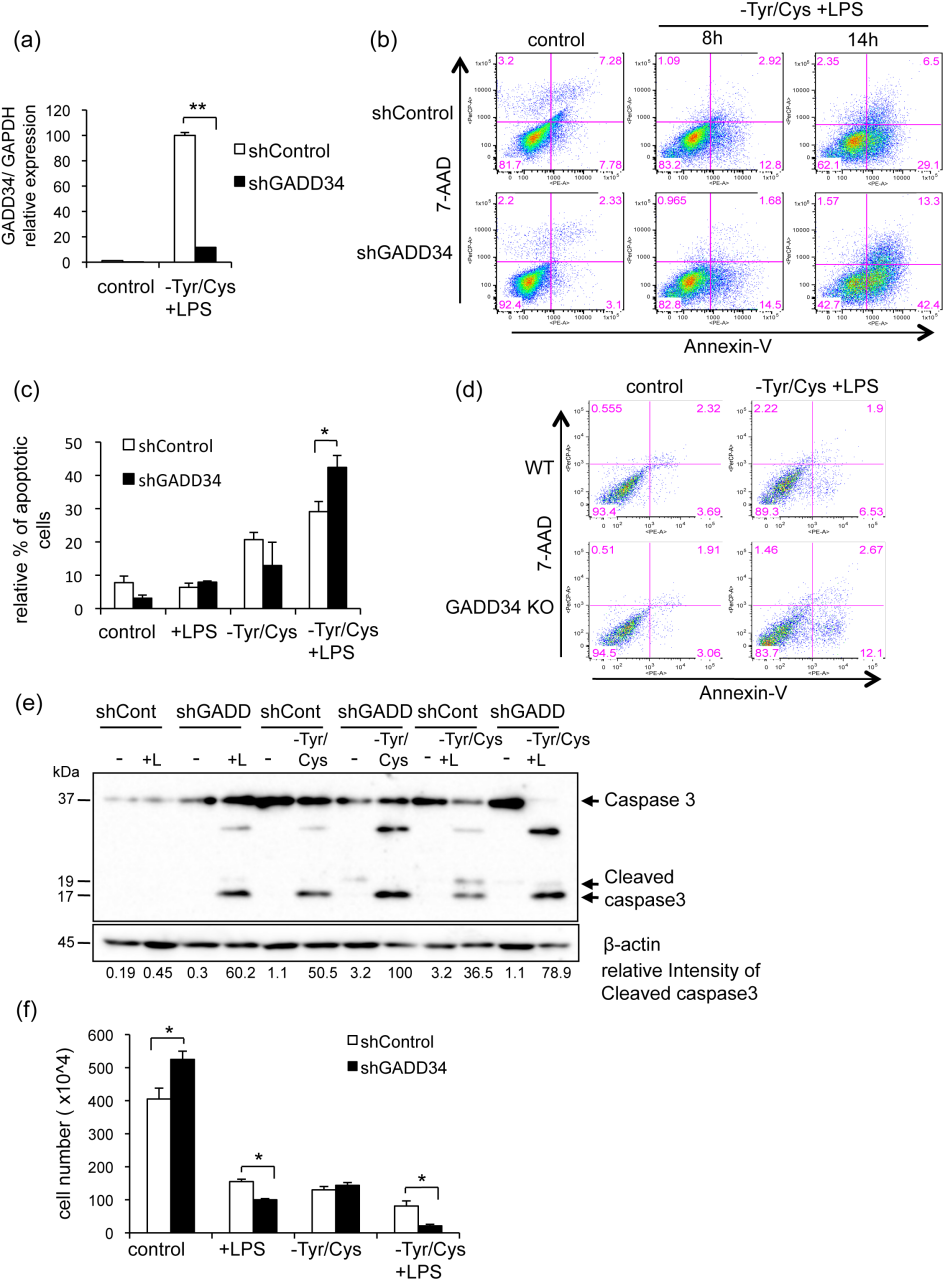
Loss of GADD34 increased cellular damage and apoptosis in RAW 264.7 cells treated with LPS and Tyr/Cys-deprivation. (a). Knockdown of GADD34 by shRNA was evaluated. Real-time PCR analysis of GADD34 expression in GADD34-deficient (shGADD34) and control RAW 264.7 cells (shControl) after treatment with LPS and Tyr/Cys-deprivation for 8 h. Graph shows the relative expression as means ± SE of three independent experiments. (b). GADD34-deficient or control RAW 264.7 cells were treated with DMEM + 10%FBS (control) or LPS (1 μg/mL) in Tyr/Cys-deprivation medium for 8 h or 14 h. Cells were stained with 7-AAD and PE-labeled Annexin V and assessed by flow cytometry. (c). GADD34-deficient or control RAW 264.7 cells were treated with LPS with or without Tyr/Cys-deprivation for 14 h. Cells were stained with 7-AAD and PE-labeled Annexin V and assessed by flow cytometry. Relative % of Annexin V^+^ 7AAD^−^ apoptotic cells shown as means ± SE of three independent experiments. (d). BMDMs from GADD34 KO or WT were treated with LPS (1 μg/mL) combined with Tyr/Cys-deprivation for 8 h. Cells were stained with 7-AAD and PE-labeled Annexin V and assessed by flow cytometry. (e). GADD34-deficient or control RAW 264.7 cells were treated with LPS (+L; 1 μg/mL) and/or Tyr/Cys-deprivation for 24 h. Cell lysates were immunoblotted with anti-caspase 3 antibody and detected full length caspase 3 (35 kDa) and cleaved caspase 3 (17, 19 kDa). (f). GADD34-deficient or control RAW 264.7 cells were treated with DMEM + 10% FBS (control) or LPS (1 μg/mL) with or without Tyr/Cys-deprivation medium for 24 h. After treatment, cells were collected and counted by trypan-blue dye exclusion using a Burker-Turk cell count chamber. Data are means ± SE of cell number from three independent experiments. Data are representative of three independent experiments (b, d, e). *p < 0.05, **p < 0.001.

In order to characterize the role of GADD34 during activation-induced cell death, we knocked down the expression of GADD34 by shRNA treatment in RAW 264.7 cells. We confirmed GADD34 knockdown by real-time PCR. The level of GADD34 expression in GADD34-deficient RAW 264.7 cells (shGADD34) was 11.6% compared to control RAW 264.7 cells (shControl) during LPS stimulation combined with Tyr/Cys-deprivation (Fig. 3a). Using GADD34-deficient or control RAW 264.7 cells, we compared the level of cell death. Annexin V^+^ 7AAD^−^ apoptotic cells were more frequent in GADD34-deficient cells than in control cells after 8 and 14 h LPS stimulation combined with Tyr/Cys-deprivation (Fig. 3b). In late stage stimulation (14 h), LPS combined with Tyr/Cys-deprivation increased the Annexin V^+^ 7AAD^−^ apoptotic cell population in GADD34-deficient cells more than that in control cells (Fig. 3b, c). When GADD34 knock out (KO) mice-derived BMDMs were subjected to the same treatment, Annexin V^+^ 7AAD^−^ apoptotic cells were elevated compared to wild-type (WT) BMDMs (Fig. 3d). The level of cleaved caspase 3 was increased by LPS stimulation in GADD34-deficient RAW 264.7 cells, but not in control cells (Fig. 3e). Tyr/Cys-deprivation with or without LPS induced the cleavage of caspase 3 in control RAW 264.7 cells. Deficiency of GADD34 permitted a greater increase of cleaved caspase 3 under these conditions. The number of GADD34-deficient RAW 264.7 cells was significantly reduced by LPS stimulation combined with Tyr/Cys-deprivation after 24 h (Fig. 3f). These results indicated that LPS combined with Tyr/Cys-deprivation initially activated macrophages, but activation was followed by cell death (activation-induced cell death). Our results showed that GADD34 suppressed activation-induced cell death.

### Effects of GADD34 on signaling of cell activation

**Figure 4:**
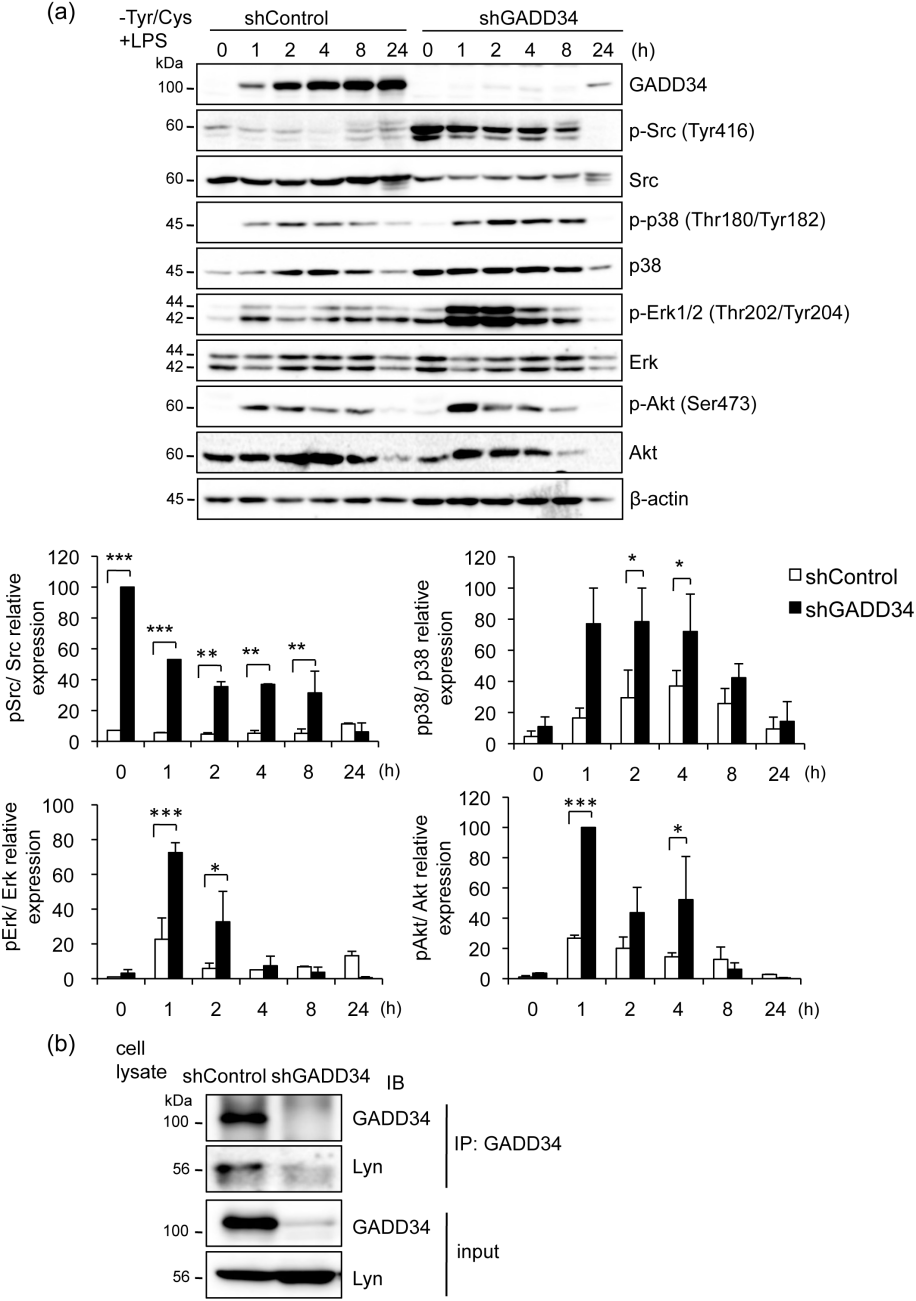
Loss of GADD34 increased cell activation signaling induced by LPS with Tyr/Cys-deprivation. (a). GADD34-deficient or control RAW 264.7 cells were treated with LPS (1 μg/mL) with Tyr/Cys-deprivation for the indicated times (0–24 h). Cell lysates were immunoblotted with the indicated antibodies. Graph shows the relative expression as means ± SE of three independent experiments. (b). Cell lysates of GADD34-deficient or control RAW 264.7 cells were subjected to immunoprecipitation (IP) using anti-GADD34 antibody. Immunoprecipitates were immunoblotted (IB) with anti-Lyn and anti-GADD34 antibodies. Data are representative of three independent experiments. The original immunoblots are presented in Supplementary Figure 4. *p < 0.05, **p < 0.01, *** *p* < 0.001.

We next analyzed the effect of GADD34 on cell activation induced by LPS combined with Tyr/Cys-deprivation in macrophage. Src family of kinases is one of important cell activation signaling. The phosphorylation of Tyr 416 in the Src-family activation kinase domain was already high in GADD34-deficient cells in the absence of stimulation (0 h). High levels were maintained by LPS stimulation combined with Tyr/Cys-deprivation (Fig. 4a). The phosphorylation of Erk and p38 mitogen-activated protein (MAP) kinase was upregulated by LPS with Tyr/Cys-deprivation. This upregulation was higher in GADD34-deficient cells than in control cells. Next we analyzed the interaction of GADD34 with Lyn, a Src-kinase family member. Immunoprecipitation with anti-GADD34 antibody confirmed the binding of GADD34 to Lyn in macrophages after LPS stimulation combined with Tyr/Cys-deprivation (Fig. 4b). These results suggested that during the activation of macrophages induced by LPS combined with Tyr/Cys-deprivation, GADD34 might regulate MAP kinase through modulating the phosphorylation of a Src family kinase, Lyn and thereby suppress cell activation.

### Effects of GADD34 on mTOR signaling and autophagy

**Figure 5:**
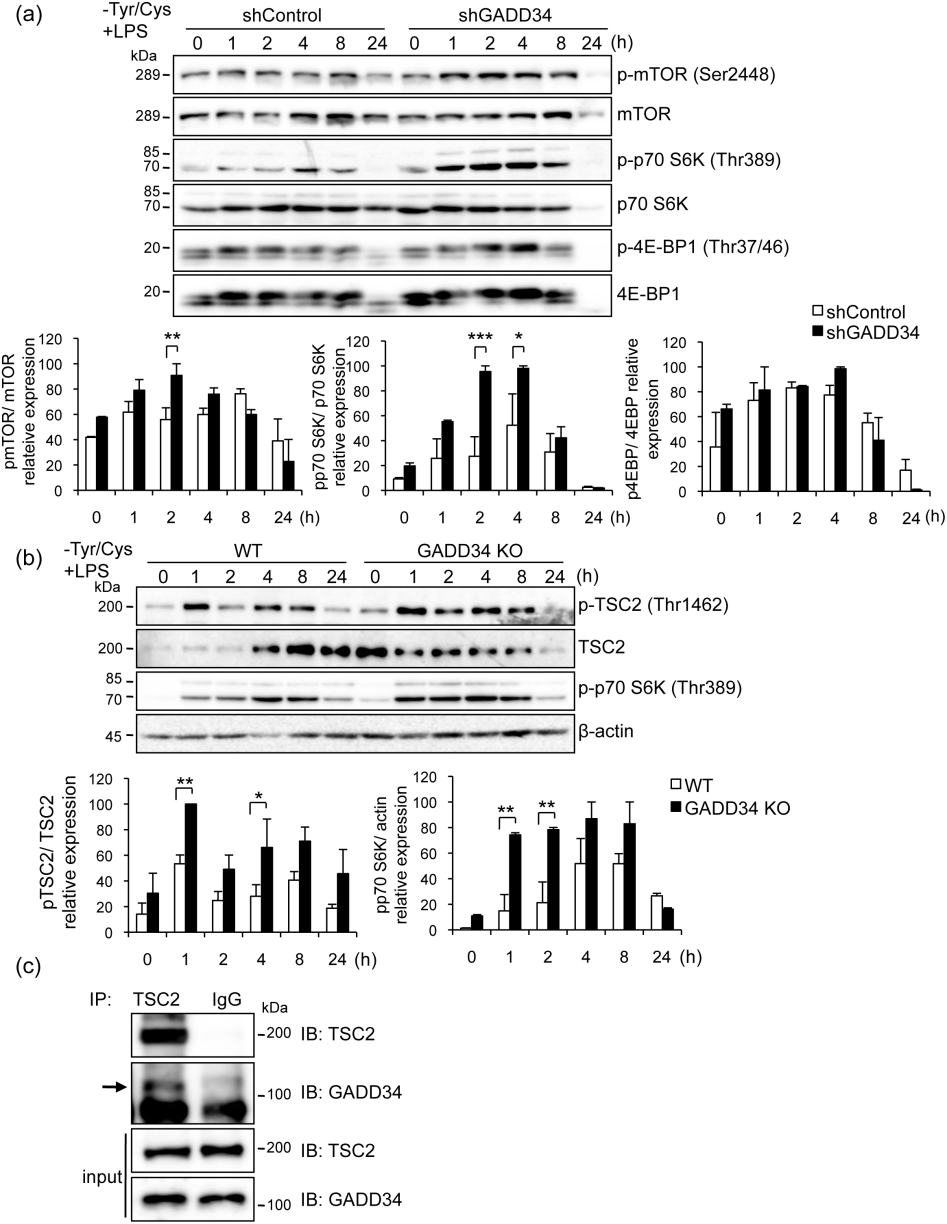
Loss of GADD34 enhanced mTOR signaling induced by LPS with Tyr/Cys-deprivation. (a). GADD34-deficient or control RAW 264.7 cells treated with LPS with Tyr/Cys-deprivation for the indicated times (0–24 h). Cell lysates were immunoblotted with the indicated antibodies. (b). BMDMs from WT or GADD34 KO mice were treated as in a. Cell lysates were immunoblotted with the indicated antibodies. (c). BMDMs from WT mice were treated with LPS (1 μg/mL) combined with Tyr/Cys-deprivation for 24 h. Cell lysates were immunoprecipitated with anti-TSC2 or IgG antibodies followed by immunoblotting with anti-TSC2 or GADD34 antibodies. Arrow indicates GADD34-specific bands. The original immunoblots are presented in Supplementary Figure 5. All immunoblots are representative of three independent experiments. Graph shows the relative expression as means ± SE of three independent experiments (a, b). *p < 0.05, **p < 0.01, ***p < 0.001.

mTOR signaling is involved in both cell proliferation and autophagy. We examined the effects of GADD34 on the mTOR signaling pathway and autophagy during stimulation by LPS combined with Tyr/Cys-deprivation in macrophages. The phosphorylation of mTOR and p70 S6K was significantly higher in GADD34-deficient RAW 264.7 cells than in control cells (Fig. 5a). The phosphorylation of 4E-BP1 was also greater in GADD34-deficient cells than in control cells. mTOR is regulated by the TSC complex, consisting of the TSC1 and the TSC2 proteins^20^. Dephosphorylation of TSC2 inhibits mTOR and activates autophagy. Using BMDMs, we analyzed the phosphorylation of TSC2 and its interaction with GADD34 during cell activation induced by LPS combined with Tyr/Cys-deprivation. The phosphorylation of TSC2 was significantly higher in GADD34 KO BMDMs than in WT BMDMs (Fig. 5b). When GADD34 KO BMDMs were subjected to LPS stimulation combined with Tyr/Cys-deprivation, the phosphorylation of p70 S6K was enhanced more than that seen in WT BMDMs. Immunoprecipitation with anti-TSC2 antibody confirmed the interaction of GADD34 with TSC2 (Fig. 5c). These results showed that in macrophages stimulated by LPS and deprived of Tyr/Cys, GADD34 suppressed the mTOR signaling pathway via interaction with TSC2 and dephosphorylation of TSC2.

**Figure 6:**
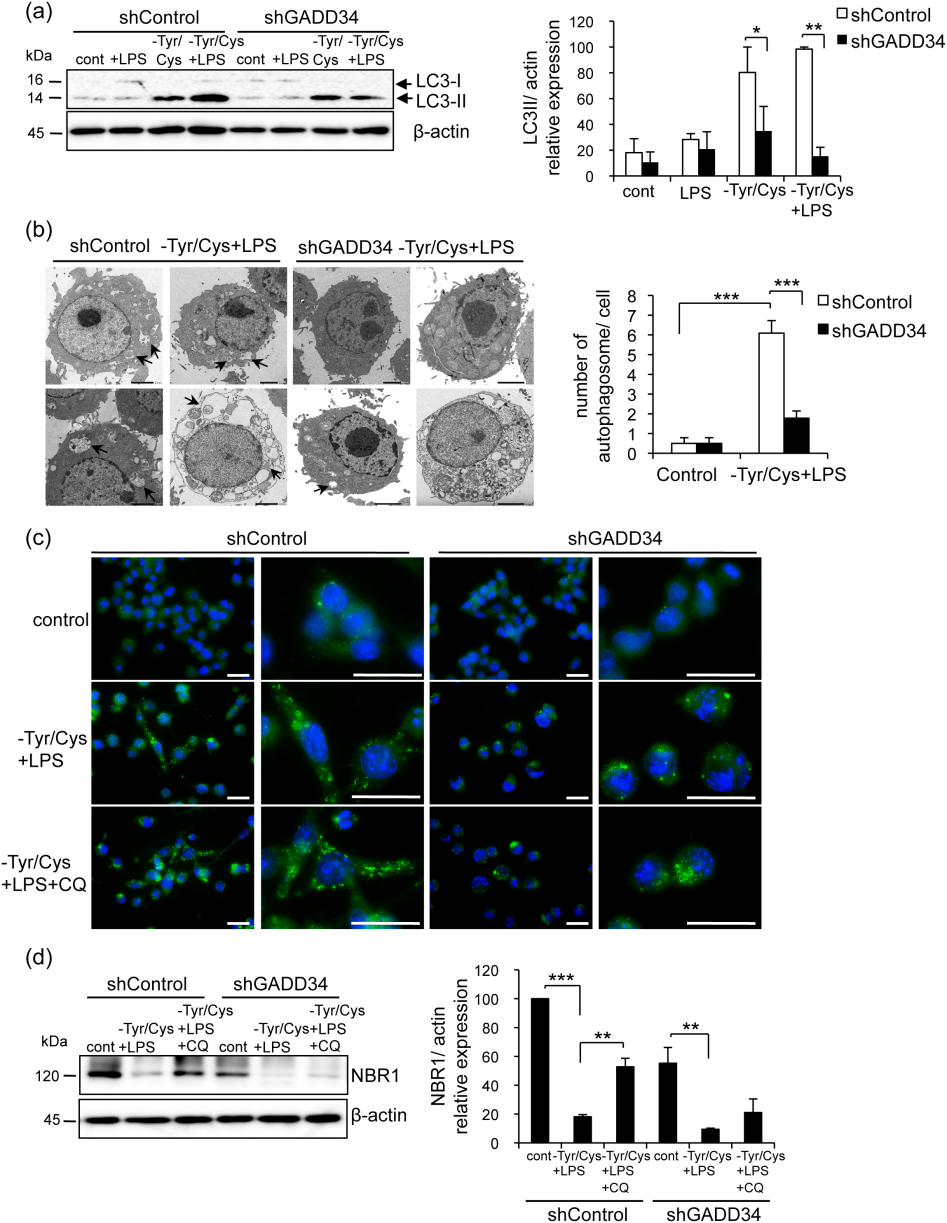
GADD34 enhanced the autophagy induced by LPS with Tyr/Cys-deprivation as detected by anti-LC3 antibody and transmission electron microscope. (a). GADD34-deficient or control RAW 264.7 cells were treated with LPS with or without Tyr/Cys-deprivation for 8 h. Cell lysates were immunoblotted with anti-LC3 and anti-β-actin antibodies. Data show means ± SE of LC3-II/β-actin expression from three independent experiments. (b). GADD34-deficient or control RAW 264.7 cells were treated with LPS combined with Tyr/Cys-deprivation for 8 h. Autophagosomes were visualized by transmission electron micrographs (TEM). Arrows indicate autophagosomes. Scale bar represents 2 μm. Data are the means ± SE of the number of autophagosome per cell from three independent experiments. (c). GADD34-deficient or control RAW 264.7 cells were treated with LPS plus Tyr/Cys-deprivation with or without 10 μM chloroquine (CQ). After 16 hrs, cells were fixed and stained with Alexa fluor 488 conjugated-anti LC3 antibody. Scale bar represents 20 μm. (d). GADD34-deficient or control RAW 264.7 cells were treated with 20 μM CQ during treatment with LPS plus Tyr/Cys-deprivation for 8 h. Cell lysates were immunoblotted with anti-NBR1 and anti-β-actin antibodies. Data show means ± SE of NBR1/β-actin expression from three independent experiments. The original immunoblots are presented in Supplementary Figure 6. *p < 0.05, **p < 0.01, ***p < 0.001.

Because mTOR signaling suppressed autophagy, loss of GADD34 might suppress autophagy in macrophages activated by stimulation of LPS combined with Tyr/Cys-deprivation. To examine this possibility, we assessed the autophagy marker LC3 during LPS stimulation and Tyr/Cys- deprivation. We found that the expression of LC3-II was lower in GADD34-deficient RAW 264.7 cells after 8 h of Tyr/Cys-deprivation with or without LPS than control RAW 264.7 cells (Fig. 6a). The presence of autophagosomes demonstrated by electron microscopy has been regarded as the standard for examining autophagy. Using transmission electron microscopy, we found that autophagic vacuoles were significantly increased in the cytoplasm of control cells stimulated by LPS combined with Tyr/Cys-deprivation compared to GADD34-deficient cells (Fig. 6b). Further, we confirmed a higher level of autophagy in control RAW 264.7 cells than in GADD34-deficient RAW 264.7 cells by assessing LC3 fluorescent puncta (Fig. 6c). LC3 fluorescent puncta was also higher in WT BMDMs than in GADD34 KO BMDMs after LPS stimulation combined with Tyr/Cys-deprivation (Supplemental fig. 2). Chloroquine (CQ) is a lysosomal inhibitor that increases intralysosomal pH and impairs autophagic protein degradation^21^. Addition of CQ increased LC3 puncta highly in control RAW 264.7 cells compare to GADD34-deficient cells (Fig. 6c). NBR1 is selectively degraded by autophagy^22^. The expression of NBR1 was decreased by treatment of LPS and Tyr/Cys-deprivation both in control RAW 264.7 cells and GADD34-deficient cells. Addition of CQ induced a greater increase in NBR1 expression in control RAW 264.7 cells than in GADD34-deficient cells (Fig. 6d). These results showed that in macrophages, loss of GADD34 suppressed autophagy that was induced by LPS-stimulation combined with Tyr/Cys-deprivation.

**Figure 7:**
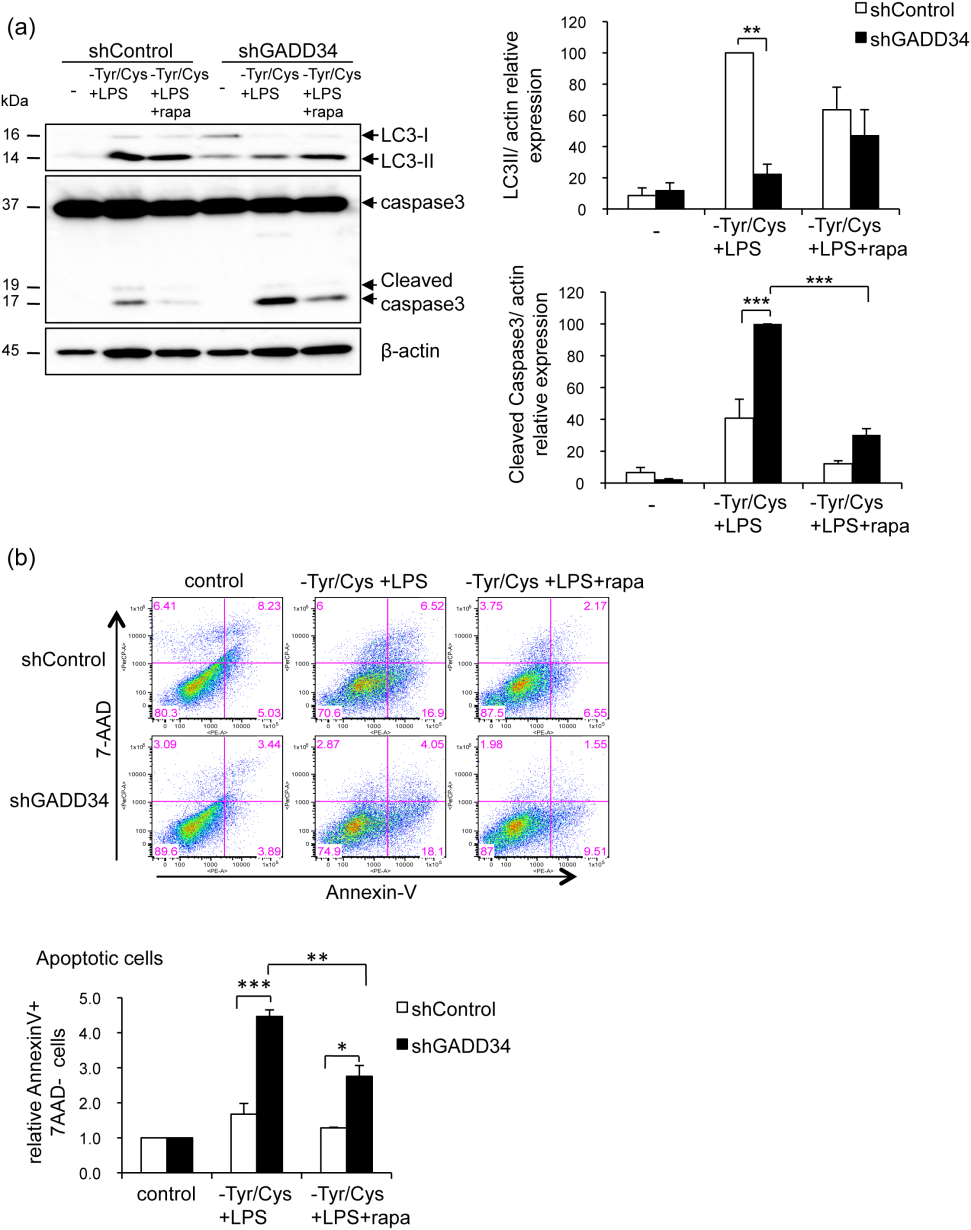
Effects of rapamycin on LC3-II expression and cleaved caspase 3. (a). GADD34-deficient or control RAW 264.7 cells were treated with rapamycin (20 nM, rapa) in LPS (1 μg/mL) and Tyr/Cys-deprivation medium for 8 h. Cell lysates were immunoblotted with the indicated antibodies. Intensities of LC3-II or cleaved caspase 3 bands were measured by densitometry as shown in graph (means ± SE). Data are representative of three independent experiments. (b). GADD34-deficient or control RAW 264.7 cells were treated as in a for 14 h. Cells were stained with 7-AAD and PE-labeled Annexin V and analyzed by flow cytometry. Graph shows the ratios of Annexin V^+^ 7AAD^−^ apoptotic cells relative to control conditions as 1 (means ± SE). Data from three independent experiments. *p < 0.05, **p < 0.01, ***p < 0.001.

Rapamycin has been shown to inhibit mTOR signaling. Addition of rapamycin to GADD34-deficient RAW 264.7 cells, which were treated with LPS and Tyr/Cys-deprivation, increased LC3-II expression and decreased apoptosis detected by cleavage of caspase 3 (Fig. 7a). Also the number of Annexin V^+^ 7AAD^−^ apoptotic GADD34-deficient RAW 264.7 cells was decreased by the addition of rapamycin to medium containing LPS and lacking Tyr/Cys (Fig. 7b). Thus, when macrophages were stimulated by LPS and deprived of Tyr/Cys, GADD34 enhanced autophagy and prevented apoptosis via suppression of mTOR pathway.

## Discussion

Here, we found that a deficiency of a specific pair of amino acids (Tyr/Cys) induced high expression of GADD34. In macrophages, GADD34 suppressed mTOR signaling and induced autophagy that resulted from exposure to LPS and deprivation of Tyr/Cys. Although deprivation of other amino acids, including Leu/Lys, Arg and Trp, also induced GADD34 expression, Tyr/Cys-deprivation was the strongest inducer of GADD34 expression in macrophages. Because tyrosine is a source of signaling molecules, Tyr/Cys-deficiency and LPS stimulation can induce macrophage cell death. GADD34-induced autophagy may be beneficial to cells, as it permits them to use their own amino acids to sustain vital functions. Inhibition of mTOR activity by GADD34 also benefits cells by reducing energy needed for cell growth under amino acid- (tyrosine) deficient conditions.

### Induction of GADD34 expression by LPS treatment combined with amino acid deprivation

Expression of GADD34 is very low under normal conditions, but it can be induced by various stresses. It has been shown that Leu-deprivation induces GADD34^19^. However, there are no reports that have extensively studied the expression of GADD34 as a function of amino acid deprivation. Here, we found that depriving macrophages of both Tyr and Cys induced GADD34 expression higher than that achieved by Leu/Lys-deprivation. GADD34 expression was further enhanced by stimulation by LPS with Tyr/Cys-deprivation. It has been shown that amino acid deprivation induced GCN2, which induces eIF2α phosphorylation and ATF4 expression^23^. Thiaville et al. examined the effect of histidine deprivation on ER stresses in HepG2 cells^24^. They found that eIF2α phosphorylation occurs through Erk phosphorylation and required GCN2 kinase activity. They found that knockdown of GADD34 had no effect on these pathways. In our study, LPS stimulation combined with Tyr/Cys-deprivation induced eIF2α phosphorylation that was enhanced in GADD34-deficient macrophages compared with control cells (data not shown).

### GADD34 suppressed cell growth and activation-induced apoptosis

We found that LPS stimulation combined with Tyr/Cys-deprivation upregulated growth-related signaling pathways and induced the expression of GADD34 in macrophages. Our experiments have shown that GADD34 suppressed a Src family kinase as well as the MAP kinase pathway during LPS-stimulation combined with Tyr/Cys-deprivation. We found that phosphorylation of a Src-family protein was higher in unstimulated GADD34-deficient RAW 264.7 cells than in control RAW 264.7 cells. We found that FBS highly stimulated Src-family phosphorylation in GADD34-deficient RAW 264.7 cells. We also confirmed that LPS stimulation without FBS induced Src-family phosphorylation. Src-family phosphorylation stimulated by LPS only was also higher in GADD34-deficient RAW 264.7 cells than in control RAW 264.7 cells (data not shown). The src family of protein tyrosine kinases includes Src, Lyn, Fyn, Yes, Lck and Hck etc and are important in the regulation of growth and differentiation of cells^25^. Lyn has been shown to interact and phosphorylate a tyrosine of Myeloid Differentiation Factor-2 (MD-2), which is an essential component of the signaling pathway following LPS stimulation^26^. Interaction of GADD34 and Lyn has been shown previously by *in vitro* gene transfection study^27^. Here we confirmed the interaction of GADD34 and Lyn by *in vivo* coimmunoprecipitation methods. Thus GADD34 might regulate phosphorylation of a src-family kinase, Lyn and consequently modulate cell growth and activation induced by LPS with Tyr/Cys-deprivation.

In the late phase of activation by LPS with Tyr/Cys-deprivation, GADD34-deficient macrophages were subject to more damage and were more apoptotic than control cells. These results indicated that GADD34 suppressed cell activation and activation-induced cell death.

### GADD34 suppressed mTOR signaling and enhanced autophagy induced by LPS activation combined with Tyr/Cys-deprivation

mTORC1 is differentially activated by distinct stimuli, including serum growth factors and amino acids^28^. Overexpression of GADD34 increases cytoprotective autophagy and cell survival mediated by the mTOR pathway in mutant huntingtin-expressing cells^29^. Suraweera *et* al. have shown that one of the mechanisms of cell death induced by proteasome inhibition is amino acid shortage^30^. They showed that cysteine rescued the cells following proteasome inhibition. Recycling amino acids is a well-established function of autophagy in starving cells^31^. Here, we showed that Tyr/Cys amino acid-deprivation coupled with LPS stimulation strongly induced GADD34 that in turn was followed by induction of autophagy through suppression of mTOR signaling. We confirmed interaction of GADD34 with TSC2 after stimulation by LPS during Tyr/Cys-deprivation in macrophages. Moreover, GADD34 suppressed the phosphorylation of TSC2. Previous reports showed that GADD34 interacted with TSC2^13^. GADD34 binds to the serine/threonine phosphatase PP1 that dephosphorylates proteins^32^. TORC1 is localized on lysosomal membranes, when cells have sufficient amino acids^33^. Upon amino acid removal, TSC2 moves to lysosomal membranes and causes TORC1 to become inactive in the cytoplasm. As a result, mTOR signaling decreases and autophagy increases. Taken together, we suggest that GADD34 in macrophages induces TORC1 release from lysosomal membranes during amino acid deprivation. This process inhibits mTOR signaling and enhances cytoprotective autophagy.

We showed that stimulation by LPS combined with Tyr/Cys-deprivation enhanced autophagy in control RAW 264.7 cells compared to GADD34-deficient cells. The increase in autophagy was demonstrated by the increase in the ratio of LC3-II/β-actin and LC3 puncta as shown by immunoblotting and fluorescence microscopy. We confirmed autophagy by electron microscopy. Furthermore CQ treatment blocked the autophagic protein degradation and enhanced NBR1 expression.

In conclusion, we have found that GADD34 was highly expressed in macrophages when exposed to LPS stimulation combined with Tyr/Cys-deprivation. GADD34 suppressed macrophage activation and activation induced-cell death by inhibiting the mTOR pathway and induction of autophagy. Macrophages stimulated by a bacterial product (LPS) might avoid excessive proliferation and cell death by upregulating GADD34, thereby supporting autophagy and the use of degraded amino acids for cell survival.

## Methods

### Mice

C57BL/6 mice were purchased from SLC Japan. GADD34 knock-out (KO) mice were established as previously described^32^. GADD34 KO mice were mated with C57BL/6 and back-crossed for more than ten generations. These mice were maintained in the Animal Research Facility at the Nagoya University Graduate School of Medicine under specific pathogen-free conditions and used according to institutional guidelines. All experiments were designed to minimize suffering. Mice were humanely sacrificed prior to Bone marrow collection. This study was carried out in strict accordance with the recommendations of the Regulations on Animal Experimentation at Nagoya University. The Animal Care and Use Committee of Nagoya University Graduate School of Medicine approved the protocol.

### Cell culture and chemicals

Murine macrophage RAW 264.7 cells and human macrophage THP1 cells were obtained from RIKEN. RAW 264.7 cells were cultured in DMEM (Sigma, D5766) supplemented with 10% heat-inactivated FBS (Equitech-Bio Inc.). THP1 cells were cultured in RPMI1640 (Sigma, R8758) supplemented with 10% heat-inactivated FBS (HyClone). Bone marrow derived macrophages (BMDMs) were prepared from GADD34 KO mice or wild-type (WT) mice. Bone marrow cells were cultured on petri dishes with RPMI1640 supplemented with penicillin, streptomycin (Gibco, 15070), 50 μM 2-mercaptoethanol, 10% heat-inactivated FBS and 10% medium conditioned by murine GM-CSF-producing Chinese hamster ovary cells, a gift from Dr. T. Sudo, Toray Silicon, Tokyo, Japan. After 7 to 10 days, floating and loosely attached cells were collected as BMDMs. LPS (L2630), rapamycin (R0395) and chloroquine (C6628) were obtained from Sigma.

### Amino acids deprivation

For amino acid deprivation experiment, basal medium was prepared from Earle's balanced salt (EBS: Sigma E2888) supplemented with MEM vitamin solution (Sigma M6895), 0.11 g/L sodium pyruvate (Sigma P4562) and 0.0001 g/L Fe(NO_3_)_3_9H_2_O (Sigma 216828). To this basal medium, we added each amino acid in the same concentration as amino acids contained in DMEM (Table 1). After we determined the maximal expression of GADD34 was achieved with Tyr/Cys-deprivation, we used Tyr/Cys-deprivation medium in DMEM, which was made by Cell Science and Technology Institute, Inc.

### Lentivirus-mediated shRNA knockdown of gene expression

The translation of GADD34 mRNA in RAW 264.7 cells was knocked down using Mission TRC mouse shRNA Lentiviral Transduction Particles (pLKO.1-puro, Sigma). The sequence of the shRNA used was as follows: CCGGGGCGGCTCAGATTGTTCAAAGCTCGAGCTTTGAACAATCTGAGCCGCCTTTTTG (shRNA TRC2, targeting exon 2) for GADD34 (shGADD34). Non-target control shRNA (Sigma, SHC 202V) was used as a control (shControl). RAW 264.7 cells were infected with viral particles and treated with 8 μg/mL polybrene (Millipore TR-1003-G), then incubated with cells for 24 h. Cells expressing shRNA were selected on 2 μg/mL puromycin (Sigma P8833) for functional studies. The extent of knockdown of GADD34 expression was confirmed by real-time PCR.

Recombinant experiments used here were approved by Committee of Nagoya University Graduate School of Medicine.

### Proliferation assay

Cells were plated (1 × 10^6^ per dish) in 6-well plates. They were cultured for 24 h and then treated with 0.25% trypsin-EDTA (Gibco, 25200-072). The number of RAW 264.7 cells was measured by trypan blue dye exclusion using a Burker-Turk cell count chamber.

### Immunoblotting

Cells were lysed in 2 × SDS-sample buffer (62.5 mM Tris-HCl pH 6.8, 2% SDS, 20% glycerol, 5% 2-mercaptoethanol, and 0.025% bromophenol blue). Protein concentrations were quantified with the Lowry assay using the RC DC protein assay kit (Bio Rad, 500-0120JA). Then, 20 μg of total protein were resolved by SDS-PAGE and transferred to PVDF membranes (Millipore, IPVH00010). Blotted membranes were incubated with 3% non-fat dry milk in PBS with 0.05% Tween-20 for 30 min to block nonspecific binding, and then the indicated antibodies were added for overnight incubation at 4°C. After 1 h incubation at room temperature with HRP-linked anti-rabbit IgG (GE Healthcare, NA9340V), protein bands were detected with ECL Prime (GE Healthcare, RPN 2232) and analyzed with LumiVision Pro (Aishin). The intensity of expression was analyzed with LumiVision Pro Analyzer 400 software (Aishin). Antibody to GADD34 (C-19, sc-825) was obtained from Santa Cruz. Anti-β-actin antibody was from Sigma (A2066). Anti-LC3 anbibody was from Novus (NB100-2220). Anti-phospho-Src family (Tyr 416, #2101), anti-Src (#2109), anti-phospho-p38 (Thr180/Tyr182, #9211), anti-p38 (#9212), anti-phospho-Erk1/2 (Thr202/Tyr204, #9101), anti-Erk1/2 (#9102), anti-phospho-Akt (Ser473, #9271), anti-caspase 3 (#9662), anti-phospho-mTOR (Ser2448, #5536), anti-mTOR (#2972), anti-phospho-p70 S6K (Thr389, #9205), anti-p70 S6K (#2708), anti-phospho-4E-BP1 (Thr37/46, #2855), anti-4E-BP1 (#9452), anti-phospho-TSC2 (Thr1462, #3611), anti-TSC2 (#4308), anti-NBR1 (#9891) and anti-GAPDH (#2118) were from Cell Signaling Technology.

### Quantitative RT-PCR

Total RNA was isolated from the cells using the RNeasy mini kit (Qiagen). Total RNA was quantified by using NanoDrop (Thermo Scientific). cDNA was then synthesized from one μg total RNA with a high capacity cDNA reverse transcription kit according to the manufacturer's instructions (Applied BioSystems). Real time PCR was performed on the MX3000P QPCR System (Agilent) using the following program: 60 sec at 95°C, followed by 40 cycles of 15 sec at 95°C, and 45 sec at 60°C. The reactions were carried out using 0.5 μL cDNA with THUNDERBIRD SYBR qPCR mix (TOYOBO). The following primers were used: murine GADD34 (forward); 5′-AGGACCCCGAGATTCCTCTA-3′, murine GADD34 (reverse); 5′-AGGTAGGGACCCAGCTTCTC-3′, murine GAPDH (forward); 5′-AACTTTGGCATTGTGGAAGG-3′, murine GAPDH (reverse); 5′-ACACATTGGGGGTAGGAACA-3′, human GADD34 (forward); 5′-GAGGAGGCTGAAGACAGTGG-3′, human GADD34 (reverse); 5′-AATTGACTTCCCTGCCCTCT-3′ and human β-actin (forward); 5′-GATGAGATTGGCATGGCTTT-3′, human β-actin (reverse); 5′-GTCACCTTCACCGTTCCAGT-3′.

### Immunoprecipitation

Cells were lysed in RIPA buffer (25 mM Tris-HCl pH8.0, 150 mM NaCl, 10% glycerol, 2 mM EDTA, 5 mM MgCl_2_, 0.5% NP40, 5 mM NaF, 1 mM Na_3_VO_4_ containing protease inhibitors (Roche, 04-693-132-001). After 20 min on ice, lysates were centrifuged for 10 min at 10,000 × g and 4°C to remove debris. Cell lysates were incubated overnight at 4°C with antibodies and protein G Sepharose (GE Healthcare, 17-0618-01). Sepharose samples were centrifuged, washed three times with RIPA buffer and one time with 0.5 M Tris-HCl pH 8.0 and boiled for 3 min with SDS sample buffer. Antibody to Lyn was obtained from Cell Signaling Technology (#2732). Anti-rabbit IgG was from Sigma (I5006).

### Immunofluorescence

Cells were cultured on cover slips (MATSUNAMI). After stimulation, cells were fixed in 100% methanol for 15 min at −20°C and blocked in 1 × PBS with 3% normal goat serum and 0.3% Triton X-100 for 1 h at room temperature. After 3 rinses in 1 × PBS for 5 min each, cells were stained with Alexa Fluor 488 conjugated anti-LC3 antibody (1:100; Cell Signaling Technology #13082) diluted in 1 × PBS with 1% BSA and 0.3% Triton X-100 at 4°C overnight. DAPI (1:1000; Sigma) was used to counter-stain nuclei. Stained cells were observed with a fluorescent microscope BZ-9000 (Keyence).

### Analysis of apoptotic and dead cells

Cells were collected and stained with PE-labeled Annexin V and 7-AAD (BD Biosciences, 559763) for 15 min. Cells were analyzed by flow cytometry on a FACS Calibur (BD Biosciences), followed by data analysis using FlowJo software (TreeStar).

### Transmission electron microscopy

Cells pellets from collected and centrifuged samples were fixed overnight at 4°C with 2.5% glutaraldehyde in 0.1 M PBS (pH 7.4) and then postfixed 1 h with 2% osmium tetroxide in 0.2 M PBS. Samples were dehydrated for 10 min in successive 50%, 70%, 80%, 90% and 100% ethanol solutions. Thereafter samples were dehydrated three times with propylene oxide for 10 min each, then infiltrated for 1 h with propylene oxide and epoxy resin (V/V = 1:1), embedded with EPON 812 epoxy resin, DDSA, NMA resin and DMP-30, and then aggregated for 48 h at 60°C. After polymerization, 70 nm ultrathin sections were made with a diamond knife using Reichert-Nissei ultracuts (Leica), and these were then stained with uranyl acetate and leas stain solution (Sigma). The stained sections were observed and photographed using a JEOL JEM-1400 EX transmission electron microscope.

### Statistical analysis

Results are expressed as means ± S.E. The statistical analysis was carried out by Student's *t*-test or one-way ANOVA followed by Fischer's PLSD test. *P* values less than 0.05 were considered statistically significant.

## Author Contributions

K.I. and S.I. wrote the main manuscript text. S.I., Y.T., R.O., K.A., S.T. and N.N. did experiments and prepared figures. All authors reviewed the manuscript.

## Supplementary Material

Supplementary Information Supplemental Figure

## Figures and Tables

**Table 1:** Concentrations of amino acids

amino acids	(g/L)	Sigma
L-Cystine.2HCl	0.0626	C2526
Glycine	0.03	G7126
L-Serine	0.042	S4500
L-Tyrosine.2Na.2H_2_0	0.1038	T1145
L-Arginine.HCl	0.084	A5131
L-Glutamine	0.584	G8540
L-Histidine.HCl.H_2_0	0.042	H8125
L-Isoleucine	0.105	I2752
L-Leucine	0.105	L8000
L-Lysine.HCl	0.146	L5626
L-Methioneine	0.03	M9625
L-Phenylalanine	0.066	P2126
L-Threonine	0.095	T8625
L-Tryptophan	0.016	T0254
L-Valine	0.094	V0500
