# Supplementary material for: GADD34 inhibits activation-induced apoptosis of macrophages through enhancement of autophagy
Source: Sci Rep. 2015 Feb 9;5:8327. doi: 10.1038/srep08327 (PMC4321179; doi:10.1038/srep08327)

Supplementary Information

GADD34 inhibits activation-induced apoptosis of macrophages through enhancement of autophagy

Sachiko Ito, Yuriko Tanaka, Reina Oshino, Keiko Aiba, Suganya Thanasegaran, Naomi Nishio, and Ken-ichi Isobe

Department of Immunology, Nagoya University Graduate School of Medicine, 65 Turumai-cho, Showa-ku, Nagoya, Aichi, 466-8550, Japan

Supplemental Figure 1.

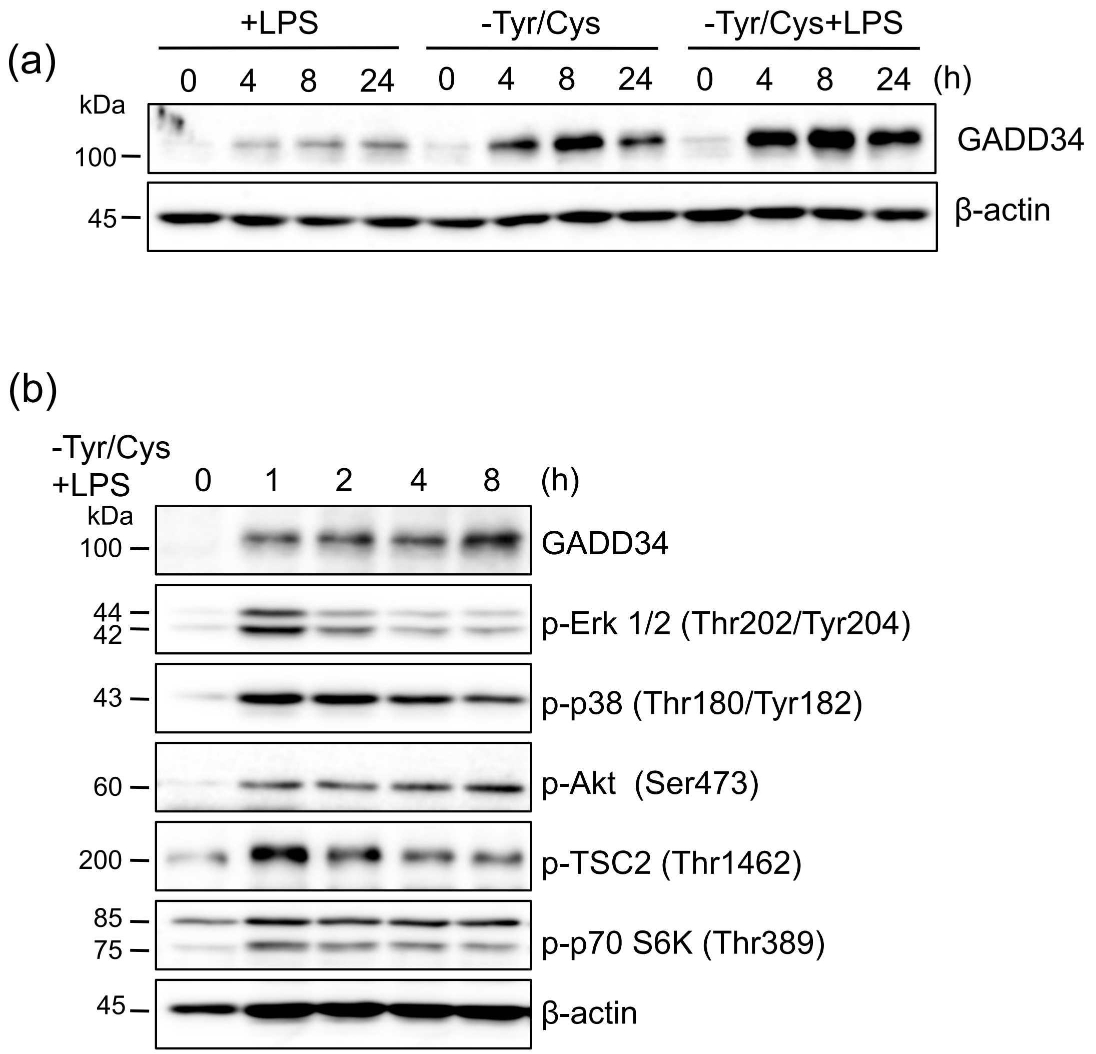

Supplemental Figure 1

(a) BMDMs (macrophage in a strict sense, which was tightly attached the petri dish) were stimulated with LPS, deprived of Tyr/Cys or both for the indicated times. GADD34 expression was determined by immunoblotting. (b) BMDMs (macrophage in a strict sense, which was tightly attached the petri dish) were treated with LPS (1 μg/mL) with Tyr/Cys-deprivation for the indicated times (0 - 8 h). Cell lysates were immunoblotted with the indicated antibodies.

Supplemental Figure 2.

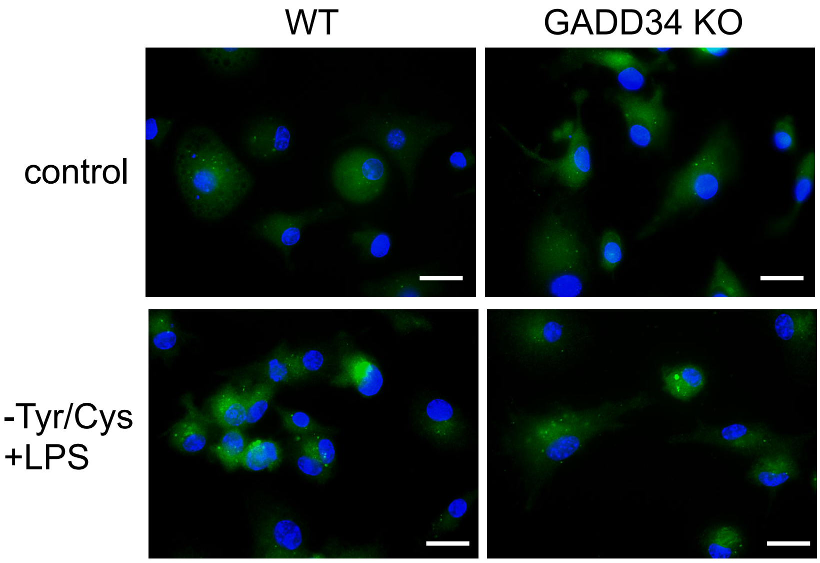

Supplemental Figure 2

WT or GADD34 KO-derived BMDMs were treated with LPS combined with Tyr/Cys-deprivation for 16 h. Cells were fixed and stained with Alexa fluor 488 conjugated-anti LC3 antibody. Scale bar represents 20 μm.

Supplemental Figure 3.

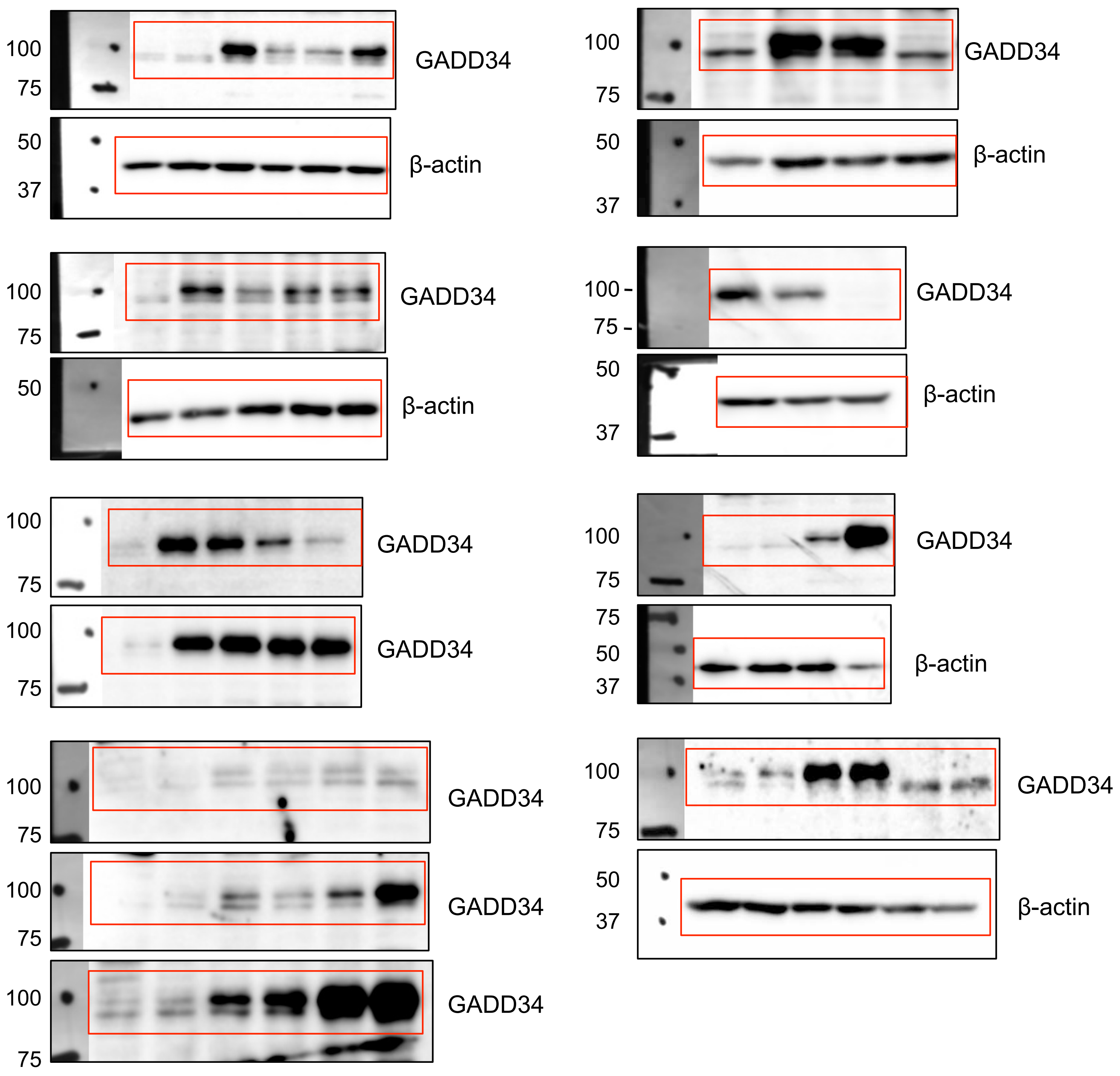

Supplemental Figure 4.

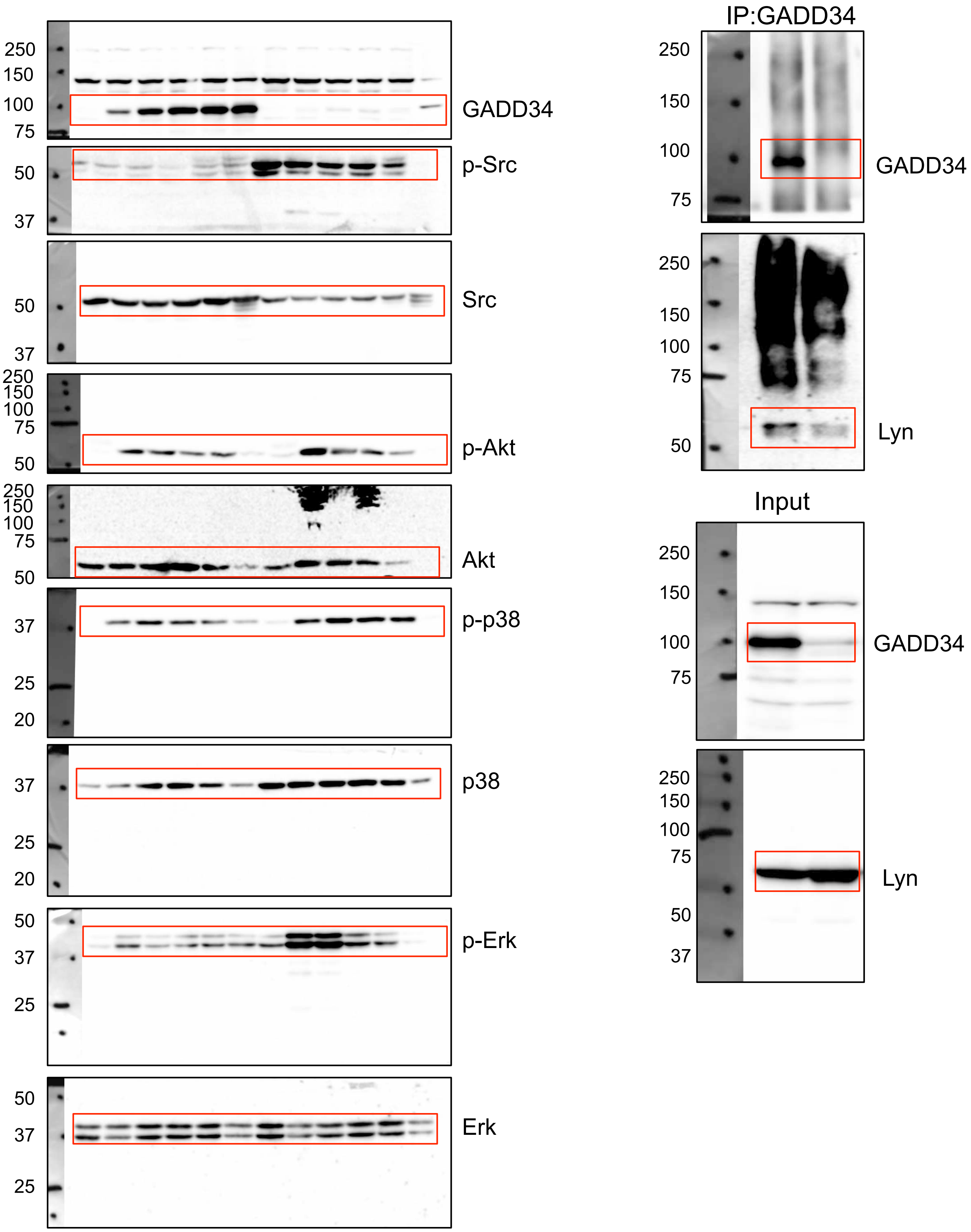

Supplemental Figure 5.

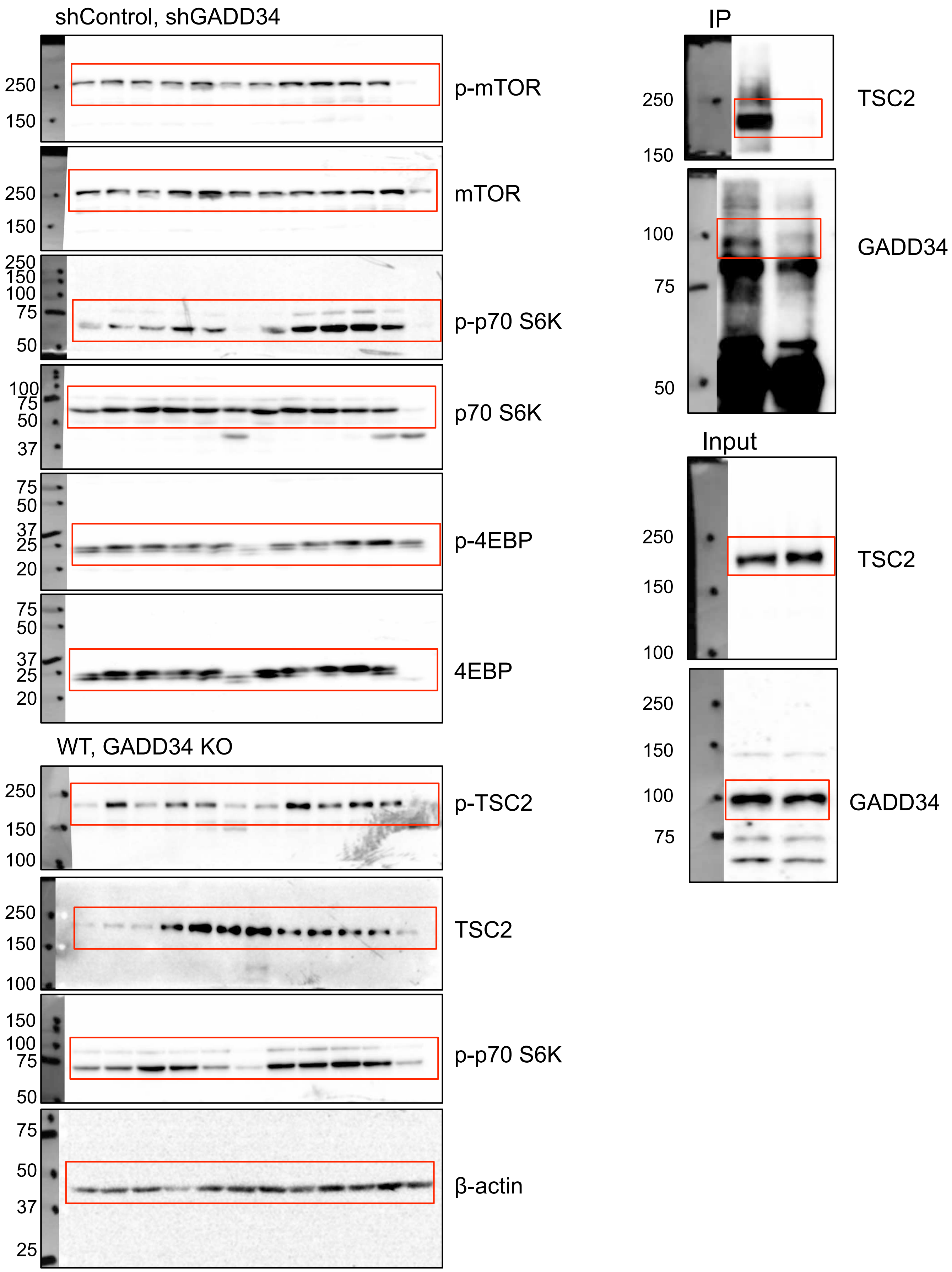

Supplemental Figure 6.

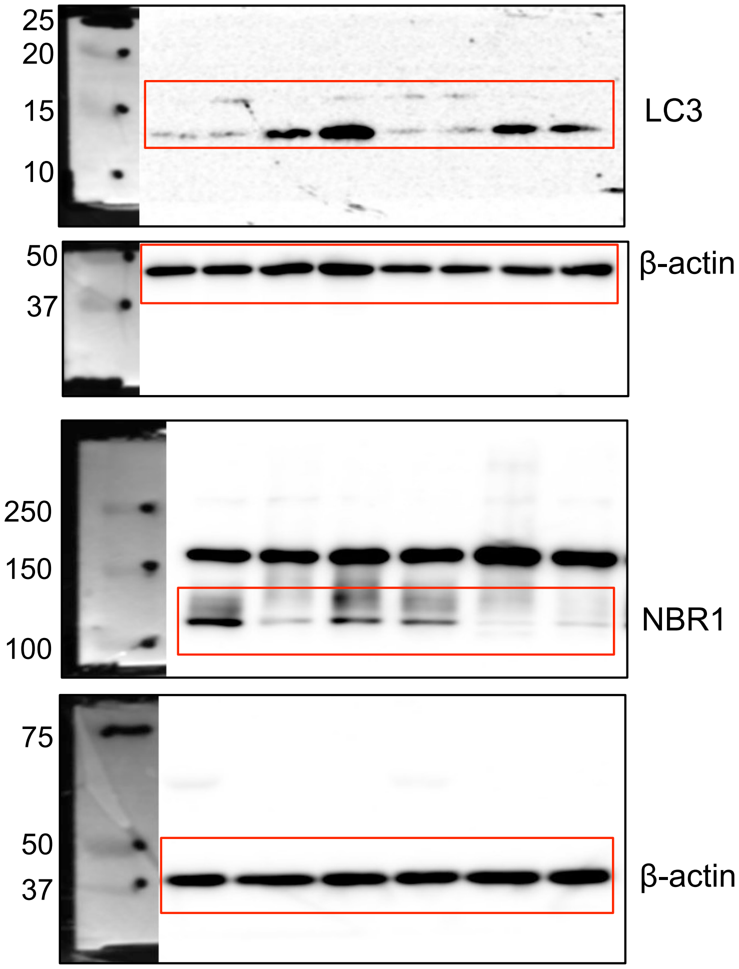

Supplement: Supplementary Information — Supplemental Figure [file srep08327-s1.pdf]
